# Phospholipidomics in Clinical Trials for Brain Disorders: Advancing our Understanding and Therapeutic Potentials

**DOI:** 10.1007/s12035-023-03793-y

**Published:** 2023-11-20

**Authors:** Mayssa Hachem, Mirja Kaizer Ahmmed, Houda Nacir-Delord

**Affiliations:** 1 Department of Chemistry and Healthcare Engineering Innovation Center, Khalifa University of Sciences and Technology, P.O. Box 127788, Abu Dhabi, United Arab Emirates; 2 Department of Fishing and Post-Harvest Technology, Chattogram Veterinary and Animal Sciences University, Chattogram, Bangladesh; 3 Riddet Institute, Massey University, Palmerston North, New Zealand; 4 Department of Chemistry, Khalifa University of Sciences and Technology, P.O. Box 127788, Abu Dhabi, United Arab Emirates

**Keywords:** Lipidomics, Brain lipid, Lipid profiling, Lipid biomarkers, Analytical approaches, Clinical trials

## Abstract

**Abstract:**

Phospholipidomics is a specialized branch of lipidomics that focuses on the characterization and quantification of phospholipids. By using sensitive analytical techniques, phospholipidomics enables researchers to better understand the metabolism and activities of phospholipids in brain disorders such as Alzheimer’s and Parkinson’s diseases. In the brain, identifying specific phospholipid biomarkers can offer valuable insights into the underlying molecular features and biochemistry of these diseases through a variety of sensitive analytical techniques. Phospholipidomics has emerged as a promising tool in clinical studies, with immense potential to advance our knowledge of neurological diseases and enhance diagnosis and treatment options for patients. In the present review paper, we discussed numerous applications of phospholipidomics tools in clinical studies, with a particular focus on the neurological field. By exploring phospholipids’ functions in neurological diseases and the potential of phospholipidomics in clinical research, we provided valuable insights that could aid researchers and clinicians in harnessing the full prospective of this innovative practice and improve patient outcomes by providing more potent treatments for neurological diseases.

**Graphical Abstract:**

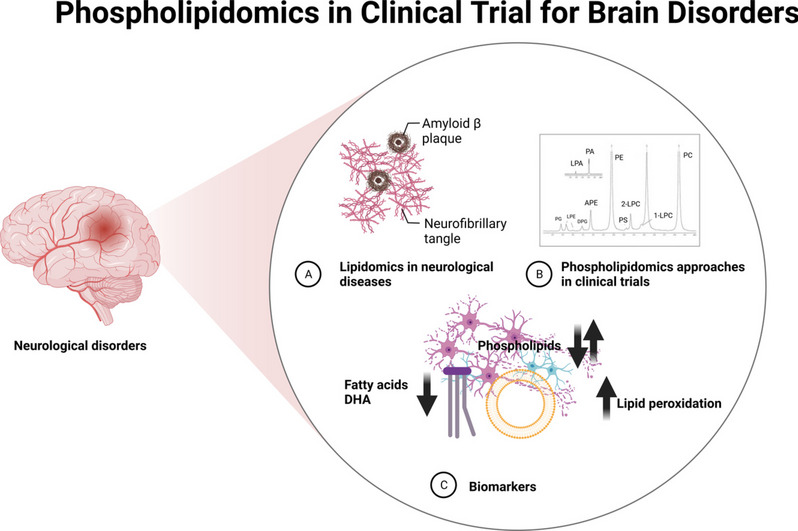

## Introduction

For decades, although the significance of lipids’ metabolism in biology and medicine was widely recognized, one of the main challenges was the lack of sensitive techniques for the identification and quantification of specific lipid species. It was not until the late 1980s that breakthroughs occurred, with individual lipid molecular species finally becoming identifiable and quantifiable through the use of soft ionization methods, including electrospray ionization (ESI) and matrix-assisted laser desorption/ionization (MALDI), respectively, by Nobel Laureates, John Fenn, and Koichi Tanaka [1, 2]. These soft ionization technologies laid the foundation for what was later known as the field of lipidomics. In the early 1990s, a variety of strategic and innovative approaches (advancement of mass spectrometry, improvement of liquid and gas chromatography) allowed the growth of the lipidomics field [3]. In 1994, a quantitative analysis of phospholipids (PLs) using ESI in both positive- and negative-ion modes was conducted by Han and Gross [4] who investigated PLs’ profiles in lipids extracted from the human erythrocyte plasma membrane and documented the influence of the dipole in the PL’s head groups and electric field–induced charge separation on ionization of several cellular polar lipids. In the same year, Kim et al. [5] reported the analysis of phospholipids (PLs) using liquid chromatography (LC) coupled with electrospray ionization-mass spectrometry (ESI-MS). During this initial period, the analysis and characterization of commercially available lipid classes, including PLs, were carried out as stated in several publications [6–9].

Later on, Kishimoto et al. [10] introduced the term “lipidome”, denoting the complete assembly of chemically diverse lipid molecular species in a cell, an organ, or a biological system. Following this, in 2003, the scope of research in lipidomics was defined by Han and Gross [11]. These researchers classified lipidomics as an emerging field greatly counting on equipment and analytical chemistry for the examination of lipid structures, abundance of isolated molecular classes, cell functions, and interfaces pinpointing the active variations of lipids throughout cellular perturbations. Therefore, lipidomics exhibited a vital role in understanding the biochemical mechanisms fundamental in lipid-connected disease procedures via the identification and quantification of variations in cellular lipid signaling, metabolism, trafficking, and homeostasis [11].

Over the past two decades, there has been significant advancement in lipidomics technologies, leading to the emergence of clinical lipidomics as a novel extension of this field. Clinical lipidomics aims to investigate metabolic pathways and networks by quantitation of the complete spectrum of lipidomes in cells, biopsies, and body fluids of patients. The results of lipidomics can be correlated with clinical proteomics, genomics, and phenomics in order to accurately identify human diseases [12, 13].

Indeed, dysregulation of lipid metabolism is meticulously allied with the initiation and progression of several diseases including cardiovascular diseases and type 2 diabetes [14] as well as several central nervous system (CNS) disorders including Alzheimer’s disease (AD), Parkinson’s disease (PD), multiple sclerosis, schizophrenia and epilepsy [15–20].

Several lipid biomarkers can be identified and quantified through clinical lipidomics. Through these lipids’ biomarkers, researchers can gain a better understanding of a disease, paving the way for the development of novel targeted therapies that can modify lipid metabolism and improve patient outcomes. Such innovative therapies hold immense promise in advancing the field of medicine and providing patients with more effective and personalized treatments.

In the present review, we focused only on clinical lipidomics mainly phospholipidomics applications in the context of CNS disorders. We discussed lipidomics in neurological diseases including lipid profiles in the brain and glycerophospholipids as biomarkers in brain disorders. Following this, we investigated the cerebral phospholipids’ content in healthy and disease conditions through the application of numerous analytical approaches such as chromatographic and spectroscopic methods.

## Lipidomics in Neurological Diseases

### Lipids’ Profiles in the Brain

Lipids are essential components of the brain and play vital roles in maintaining cerebral structure and biological functions. The brain contains approximately 60% of fats [21]. To examine the lipid content in the brain, researchers investigated several model systems including rodents [22, 23], tissue cells [24, 25], or even post-mortem human brain tissues [26–29]. Among these lipids, Glycerophospholipids (GPLs), the furthermost lipid class in the brain, serve as primary building blocks of a cell’s membrane [30]. The composition of a cell’s membrane is essential for various cellular functions including ion channels’ regulation, neurotransmitter transport, and signal transduction [31]. Additionally, GPLs are involved in myelin formation in charge of insulating nerve fibers, allowing a fast and efficient transmission of electrical impulses [32].

**Fig. 1:**
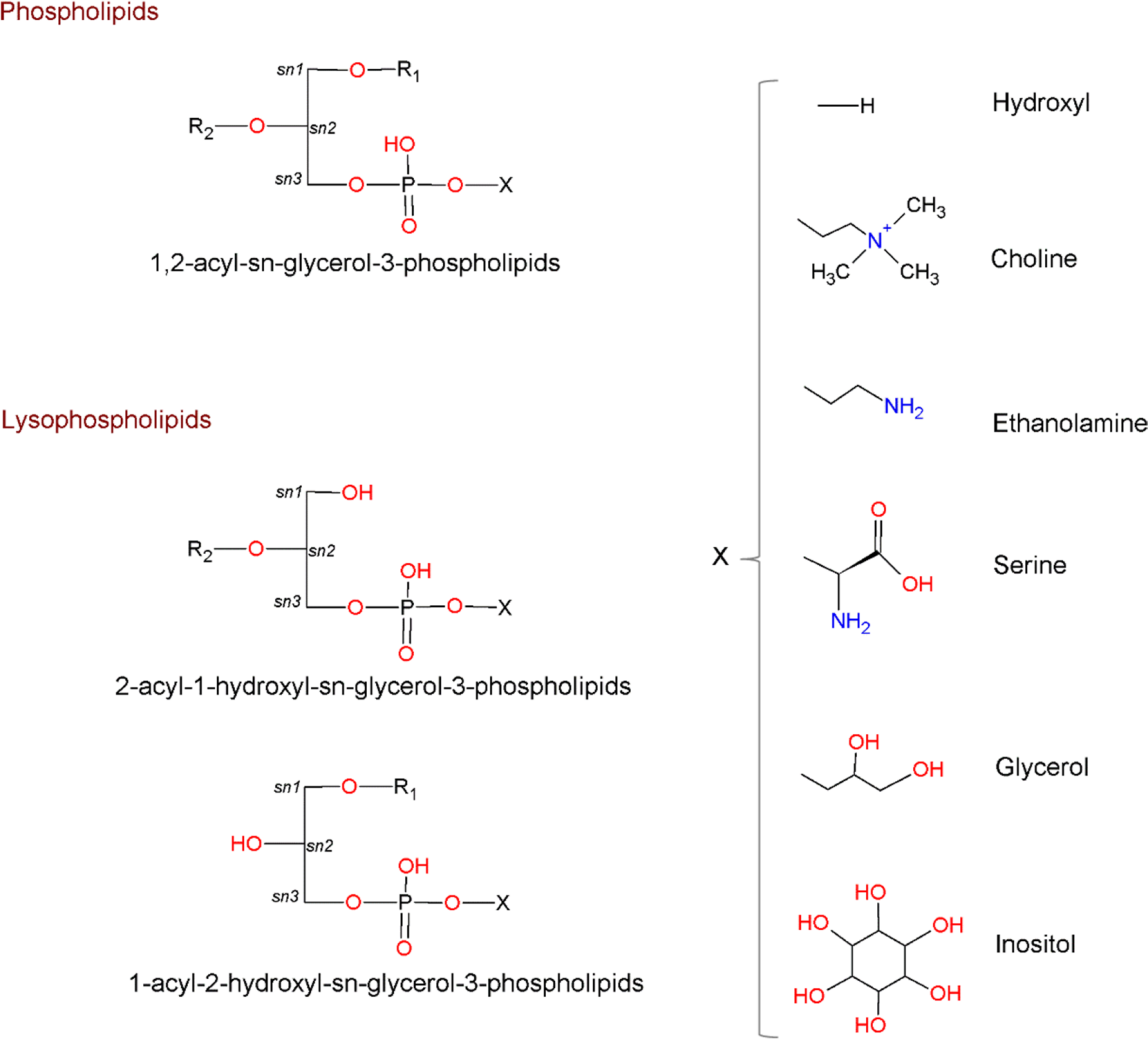
Overview of glycerophospholipids structures. Phospholipids contain two FAs esterified to a glycerol backbone at *sn-1* and *sn-2* positions, whereas lysophospholipids have only one FA esterified at *sn-1* or *sn-2* position. For all glycerophospholipids species, the phosphate group is located at *sn-3* position. The polar group differs according to the X group (X corresponds to one of the following: hydrogen, choline, ethanolamine, serine, glycerol, or inositol)

As regards of GPLs’ chemical structure, GPLs have an amphiphilic structure with a hydrophilic polar head and a hydrophobic moiety “tail”. This specific structure provides to GPLs a key function in cell structure and metabolism. As summarized in Fig. 1, GPL classes differ according to polar head at *sn-3* position forming different classes of GPLs such as phosphatidylcholine (PC), phosphatidylethanolamine (PE), phosphatidylserine (PS), phosphatidylinositol (PI), phosphatidylglycerol (PG), and phosphatidic acid (PA) [33]. In the brain, high content of omega-3 polyunsaturated fatty acids (PUFA) such as docosahexaenoic acid (DHA) and eicosapentaenoic acid (EPA) are primarily esterified in different GPLs such as PC, PE, and PS [34].

Additionally, bioactive lipids, such as lysophospholipids (LysoPLs), are generated through the action of phospholipase A_1_ (PLA_1_) and/or phospholipase A_2_ (PLA_2_) on GPLs [35] as illustrated in Fig. 1. LysoPLs are characterized by the presence of a single fatty acid (FA) at either *sn-1* or *sn-2* position and play vital roles in signaling cascades and as mediators of FAs across cell membranes [36, 37]. LysoPLs can act as signaling molecules regulating cell growth, differentiation, and apoptosis [33]. Additionally, they are involved in the regulation of synaptic plasticity and neuroinflammation in the brain [38]. In the brain, specific LysoPLs including lysophosphatidylcholine (LysoPC), lysophosphatidic acid (LysoPA), lysophosphatidylinositol (LysoPI), and lysophosphatidylserine (LysoPS) have been identified as important bioactive lipids [33]. More importantly, LysoPC acts as a carrier for DHA to the brain, where it is transported via a specific receptor/transporter named Mfsd_2_a (major facilitator superfamily domain containing 2A) located in the endothelial cells of BBB [39–41].

In addition to GPLs and LysoPLs, the human brain contains a complex mixture of essential lipids including sphingolipids and cholesterol [42, 43]. Indeed, sphingolipids are involved in cell signaling and regulation of specific enzymes’ activity in addition to other critical roles in CNS including dendritic development and aging [44–46]. Whereas cholesterol, found in high concentrations in the brain, is fundamental for the membrane’s structure and function [47, 48].

In humans, during the aging process, changes in lipids composition of the post-mortem brain region were investigated [49, 50]. Several autopsied brains of middle-aged and elderly (40 to 80 years old) with 17 cases including 12 healthy males and 7 females were examined. Lipids were isolated from various regions including the olive, upper vermis, substantia nigra, thalamus, hippocampus, putamen, caudate, occipital cortex, parietal cortex, entorhinal cortex, and frontal cortex. Extracted lipids were analyzed through gas chromatography with flame-ionization detection (GC-FID). Comparing the fatty acid (FAs) composition of the middle-aged brain to that of the elderly showed a well-preserved lipid profile. Minor changes in FA chain length, high monounsaturated fatty acids (MUFAs) content, and PUFAs (omega-6 and omega-3) predominance in the inferior temporal cortex and cingulate gyrus involved in memory were observed [49, 51].

### Glycerophospholipids as Biomarkers of Brain Disorders

In the brain, when comparing the lipid profiles of healthy people to unhealthy, several alterations were observed [16]. Several researchers suggested that alterations in the composition of cerebral lipids have been linked to various neurological conditions [33, 52] including AD and PD [16, 48, 50]. For example, researchers have identified alterations in the levels of PC, sphingomyelin, and ceramides in the brain as early biomarkers of AD [27]. Moreover, LysoPL dysregulation has been associated with the development and progression of several neurological disorders [38, 53]. Therefore, identifying potential therapeutic interventions that target LysoPLs became an active area of research.

Indeed, it is interesting to note that researches on post-mortem brain biopsies of individuals with neurodegenerative diseases showed a significant variation in lipid composition, particularly with regard to unsaturated and saturated fatty acids [54]. In Particular, PD and AD diseases are associated with a significant increase in unsaturated fatty acids (USFA) compared to saturated fatty acids (SFA). Up to 80% of lipids in brains affected by PD and AD contained USFA, while only 20% enclosed SFA [36]. These results suggested that USFA may play a significant role in the pathology of these diseases, possibly by affecting the structure and/or function of cell membranes in the brain. This may also have implications for understanding the progression of these diseases and developing new treatments for individuals affected by them.

In fact, this predominance of USFA may be a consequence of brain disease to prevent brain tissue destruction. PUFA, especially omega-3 fatty acids such as alpha-linolenic acid (ALA), generally serves as a precursor for the synthesis of various anti-inflammatory molecules including EPA and DHA, produced through a series of enzymatic reactions from ALA and is known to exhibit strong anti-inflammatory effects in the body. Therefore, these biomolecules potentially protect the brain from inflammation and degeneration in addition to their neuroprotective effects [37]. One of the specialized pro-resolving lipid mediators derived from DHA called neuroprotectin D1 (NPD1) showed the capability to limit the formation of amyloid plaques associated with the neurons’ degeneration in AD [55, 56]. Indeed, NPD1 exhibited potent anti-inflammatory and neuroprotective effects in several neurological conditions. Its mechanism of action involved promoting neuronal survival, reducing inflammation, and inhibiting apoptosis. Moreover, NPD1 modulated various signaling pathways in the brain, including those involved in synaptic plasticity and neurogenesis [57].

#### Alzheimer’s Disease

AD is the furthermost prevalent neurodegenerative disease and cause of dementia [58]. In AD patients, the alterations in the lipidome and the way they relate to AD are poorly understood. Nevertheless, changes in lipid metabolism have been stated to play a significant role in AD pathogenesis associated with the accumulation of β-amyloid (Aβ) plaques and neurofibrillary tangles in the brain [59, 60]. Furthermore, through lipidomics analysis, researchers identified changes in various lipid classes including FAs, PLs, and SPs in the brains of AD patients [61]. Additionally, in these patients, the levels of cerebral ceramides in the brain, involved in the formation of β-amyloid (Aβ) plaques, have increased [62]. Moreover, PC and PE levels, in the brain, were significantly declined and PLs deacylation products glycerophosphocholine were improved in the frontal, primary auditory, and parietal cortices [63]. A significant decrease in phosphatidylcholine-plasmalogen (PC-PL) was observed in the frontal cortex of the brain. Indeed, PC-PL influences γ-secretase activity responsible for amyloidogenic processing the cause of AD [51, 64].

Through lipidomics studies, in AD patients, specific and detailed lipid profiles characterized by the enrichment of SFA and PUFA in neutral lipids (DAG 28:0, TAG 58:10, and TAG 48:4), polar lipids including PLs (PE 36:1, PC 40:6, PS 36:3, PS 36:6) and sphingolipids in lipid rafts with a low level of LC-PUFA such as DHA were obtained [50, 65, 66]. These results are favorable for suggesting a new treatment targeting lipids’ metabolism as a complementary or alternative therapy for AD.

#### Parkinson’s Disease

PD is the second common neurodegenerative disease occurring in people over 60 years of age [67]. Multiple studies applying lipidomic tools have demonstrated significant changes in the levels of various lipid classes in the brains of PD patients [26]. Alterations in GPLs, SPs, and cholesterol esters have been detected in PD patients, suggesting their potential role in the pathogenesis of the disease. More specifically, PLs and SPs were the most abundant lipids with saturated or unsaturated species including PC 36:3, PE 36:2, PI 34:2, PS 36:3, and SM 18:1 [68]. Furthermore, in the brains of PD patients, researchers have revealed an increase in lipid peroxidation, a process that leads to oxidative damage of lipids [69–71], resulting in the production of reactive oxygen species (ROS). The latter is known to contribute to neuronal damage and degeneration in PD [72–74].

Lipidomics has emerged as a precious tool in PD research, offering crucial insights into the role of lipids in diseases’ pathogenesis. Through the identification of specific lipids altered in PD patients, lipidomics can provide new targets for developing novel therapeutic interventions. Thus, lipidomics has the potential to open up new avenues for developing more effective PD treatments [68].

#### Neurotoxicity in the Brain

Furthermore, lipid profiling is supportive in investigating the brain neurotoxicity effect in case of disorders. As reported in anesthetic neurotoxicity cases as well as chronic alcohol, human brain alteration was observed through lipid biomarkers characterized by mass spectrometry [75, 76]. In neurotoxicity, LC-PUFAs were the main lipid biomarkers with a notable increase in the prefrontal cortex and striatum region. In chronic alcohol cases, lipid alterations caused by alcohol could be due to the endoplasmic reticulum’s (ER) stress response to the cerebral lipids’ alteration [76].

#### Multiple Sclerosis

Multiple sclerosis is a chronic autoimmune disease affecting the CNS. In multiple sclerosis patients, lipidomics has investigated the lipids’ profile and alterations in the brain of these patients compared to healthy individuals. Several modifications in the lipid metabolism of FA, ceramides, and PLs were observed in addition to lipid peroxidation products. Furthermore, a decrease in plasmalogens and an increase in the sphingolipids’ level in the cerebrospinal fluid of multiple sclerosis patients were observed [77, 78]. Moreover, lipidomics has revealed changes in GPLs, SPs, and cholesterol in the blood of these patients, suggesting that lipid metabolism is dysregulated in multiple sclerosis disease [79]. When researchers established a phospholipidome signature in the serum of these patients [80], significantly low levels of specific PLs including PE, PC, LysoPE, ether-linked PE, and ether-linked PC species were detected [80]. Plasmalogens PC and PE, natural endogenous antioxidants as well as PC and PE with PUFA esterified classes in comparison to healthy controls PC 34:3, PC 36:6, PE 40:10, and PC 38:1 may be appropriate as biomarkers for medical applications in multiple sclerosis [80]. These findings may allow better understanding of the pathogenesis of multiple sclerosis and may lead to the development of new diagnostic and therapeutic strategies.

#### Psychosis

In addition to all previous diseases, in the context of psychosis, a low proportion of EPA (29%), DHA (27%), and AA (16%) were observed in the patients’ brain in comparison to healthy people in addition to high concentrations of SM (16%) and lower concentration of PE (46%) compared to the control group [81]. These results suggested that a decrease of PUFA accretion to the brain, mainly omega-3 PUFA, can be either the consequence or cause of psychosis in subjects.

#### Schizophrenia

Lipidomics has also been used to investigate the potential role of lipids in Schizophrenia, a complex and severe mental disorder characterized by a range of cognitive, emotional, and behavioral symptoms. Several studies have revealed alterations of FA, PC, and ceramides in brain tissues mainly the prefrontal cortex, grey and white matter. Significant alterations of SFA and MUFA (16:0, 18:0, 18:1) and PUFA (22:5, 22:6) in total lipids, TGs or PLs were observed. Around 20 PC species with SFA and PUFA and only three ceramide species were observed with MUFA mostly [18, 82]. However, Taha et al. [83] in 2013 concluded that total lipid, phospholipid, plasmalogen, triglyceride, and cholesteryl ester concentrations did not differ significantly between schizophrenia and control subjects. Only cholesteryl esters could be considered as a potential biomarker in the prefrontal cortex in schizophrenia. These findings propose that lipid metabolism may be dysregulated in schizophrenia and may contribute to the physiopathology of this disorder. However, further studies are needed to elucidate the underlying mechanisms and to determine the potential diagnostic and therapeutic implications of these results.

#### Epilepsy

Cerebral lipids’ composition in patients with neurological disorders characterized by recurrent seizures as epilepsy, showed a specific profile in the temporal lobe (hippocampus), and alterations in PC and PE content were detected [84, 85]. These variations may be related to the primary mechanisms of epilepsy, including inflammation, oxidative stress, and changes in neuronal membrane function [86]. These lipids may play a role in the development and progression of epilepsy [84]. Finally, lipidomics may be helpful in understanding the pathophysiology of epilepsy and may lead to the identification of novel therapeutic targets for this disorder.

Altogether, these findings highlight the importance of investigating the brain’s lipids as potential biomarkers for early diagnosis and treatment of neurological disorders. Through the identification of changes in brain lipids, researchers could be potentially able to develop new diagnostic tools and treatments for neurodegenerative diseases. Along with genomics and metabolomics, these observations could serve as powerful tools to elucidate the brain’s functions and tackle brain diseases by suggesting novel treatments [87–89].

Finally, the profile of lipid species regarding their total chain length and number of double bonds in human brain disorders (Parkinson’s disease, Alzheimer’s disease, Schizophrenia/bipolar disorder, multiple sclerosis, epilepsy) were summarized in Table 1. As illustrated, several lipid species such as Bis(monoacylglycero)phosphate (BMP), PC, PE, PS, PI, PG, SM, diacylglycerol (DAG), triacylglycerol (TAG), ceramide (Cer), Lyso-PC with different FA chain lengths, and a number of unsaturated content are considered as biomarkers.

**Table 1:** Overview of lipid biomarkers for several brain disorders

Diseases	Model system and size of study	Brain region	Lipid biomarkers	Outcome	References
Alzheimer’s disease	Post-mortem human brain tissue Sample size is 10 for three categories	Hippocampus, frontal cortex, entorhinal cortex	DAG 28:0; TAG 58:10; TAG 48:4; PE 36:1; PC 34:1; PC 36:1; PC 36:2; PC 36:4; PC 38:4; PC 40:6; PS 36:3; PS 36:6; PS 38:5; TAG 22:	Lipid rafts in AD brains have abnormal lipid profiles compared to healthy brains Lipid profiles in AD brains show lower levels of n-3 long-chain polyunsaturated fatty acids (such as DHA, 22:6n-3) and monounsaturated fatty acids (such as 18:1n-9, oleic acid) There is a decrease in unsaturation and peroxidability indexes in lipid rafts from AD brains	[50, 65]
Parkinson’s disease	Post-mortem human brain (10 control samples without PD and 10 samples diagnosed with PD)	Substantia nigra (SN)	BMP 42:8; PC 36:3; PE 36:2; PI 42:10; PI 34:2; PS 36:3; SM 18:1; SM 14:0; Cer 24:1; Cer 18:0	Lipid analysis of SN (substantia nigra) indicated the presence of a neuroinflammatory element, with notably increased levels of the endosomal lipid Bis(monoacylglycero)phosphate 42:8. Additionally, among the lipids found in the putamen, two out of three showed a depletion of saturated sphingomyelin species	[68]
Schizophrenia/bipolar	Forty-five human subjects including 15 schizophrenia, 15 bipolar (3 off medication at the time of death), and 15 control individuals	Prefrontal cortex (Brodmann area 9)	PC 34:1; PC 38:6; PC 40:7; PC 36:4; PC 34:2; PC 38:5; PC 38:4; PC 36:1; PC 36:2; PC 35:1; PC 33:1; PC 35:2; PC 32:0; PC 36:4; PC 34:5; PC 38:2; PC 30:0; PC 40:6; PC 34:0; Cer 36:2; Cer 36:1; Cer 34:1	Gray matter phosphatidylcholine levels were affected by antipsychotic medication, whereas white matter phosphatidylcholine levels were not influenced Changes in free fatty acids and ceramides were not observed in either white or gray matter because of antipsychotic treatment Overall, the study suggests that lipid abnormalities may be inherent features of both schizophrenia and bipolar disorder	[82]
Multiple sclerosis (MS)	Multiple sclerosis (MS) patients (*n* = 9) and control samples with samples of non-MS individuals (*n* = 9)	Central nervous system	C16:1; C18:4n-3	Lipid peroxidation is increased in CSF, indicating oxidative stress Specific protein modifications linked to lipid peroxidation were observed in CSF proteins There are elevated autoantibodies against lipid peroxidation-modified proteins in CSF These results suggest that autoimmune responses to lipid peroxidation may be a significant factor in MS	[90]
Epilepsy	Post-mortem brain samples from 15 epilepsy-diagnosed people (ENOS) and 15 control samples	Frontal cortex (BA8/46	LysoPC 16:0; LysoPC 18:1; PC 44:2; PE 43:1; PG 18:1/23:2; PS 21:0/18:0	Increased lipid peroxidation in post-mortem brain from epilepsy patients Butyrate metabolism was the major biochemical pathway found to be significantly perturbed in the post-mortem epileptic brain	[84, 85]

*BMP* Bis(monoacylglycero)phosphate, *PC* phosphatidylcholine, *PE* phosphatidylethanolamine, *PS* phosphatidylserine, *PI* phosphatidylinositol, *PG* phosphatidylglycerol, *SM* sphingomyelin, *DAG* diacylglycerol, *TAG* triacylglycerol, *Cer* ceramide, *Lyso-PC* lysophosphatidylcholine

## Phospholipidomics Approaches in Clinical Trials

Phospholipidomics is a subclass of lipidomics focusing on the characterization and quantification of various PLs. Phospholipidomics studies have been carried out in clinical trials to identify potential biomarkers and therapeutic targets for various diseases [91, 92]. These studies have involved the analysis of PLs, including their structure, composition, location, concentration, and function in biological samples.

Several analytical approaches were developed to detect and quantify PL classes in clinical trials. Indeed, researchers emphasized the importance of chromatographic techniques in phospholipidomics for investigating clinical trials including Thin-Layer Chromatography (TLC), high-performance liquid chromatography (HPLC), and gas chromatography (GC) [93]. TLC, GC, and HPLC are commonly employed in phospholipidomics. These techniques allow the separation and quantification of complex lipid mixtures. In addition to chromatography, spectroscopic methods such as nuclear magnetic resonance (NMR) spectroscopy, Raman spectroscopy, Fourier-transform infrared spectroscopy (FT-IR) as well as mass spectrometry (MS) approaches like electrospray ionization (ESI-MS), matrix-assisted laser desorption/ionization (MALDI), and isotope-ratio mass spectrometry (IRMS) were also effective for lipids analysis within this context [93]. These techniques offer unique strengths, such as the ability to probe molecular structures and detect subtle changes in lipid conformation. Each of these analytical approaches has its own advantages and limitations, making them useful for different applications. Table 2 represents the summary of different chromatographic and spectroscopic techniques commonly used in phospholipidomics.

**Table 2:** Summary of different chromatographic and spectroscopic techniques commonly used in phospholipidomics

Analytical approaches	Description	Advantages	Disadvantages	Reference
Thin-layer chromatography (TLC)	- Widely used earliest qualitative techniques for separating lipid class - TLC relies on the differential movement of compounds through a thin layer of adsorbent material, typically silica gel or alumina applied to a glass or plastic plate in a mobile liquid phase (a mixture of organic solvent and water)	- Cost-effective - Simple and easy to run - No instrument is required - Requires a few ng of sample - Able to identify minor impurities - Suitable for obtaining a quick result on lipid class - High versatility as it can be used in different solvent systems and various stationary phases - No complex data analysis is required to understand the TLC result	- The produced band has limited resolution, making it less suitable for the identification of closely related compounds - Long-term storage of TLC plates resulted in lipid oxidation - Slow process, low accuracy, and less reproducibility - The stationary phase completely absorbs the samples, leading to inaccurate results - TLC is often manual and can be less efficient for high-throughput analysis than GC or HPLC techniques	[94, 95]
Automated high-performance thin-layer chromatography (HPTLC)	- An advanced form of TLC that offers superior separation using optimized coating material - Mostly used for phytochemical analysis - Provide both qualitative and quantitative result	- Offers better separation of molecules thanks to TLC - Higher resolution compared to the traditional TLC method - More reproducible and accurate than TLC	- Requires organic solvents with high polarity - Less accurate in quantification compared to HPLC - Less flexibility in changing running conditions than TLC	[96, 97]
Thin-layer chromatography—flame ionization detection latroscan system (TLC-FID)	- Equipped with a latroscan detection system, which combines the resolution efficacy of TLC and FID	- Combined with FID, TLC is useful for quantifying PLs and LysoPLs - Provides better resolution and sensitivity than traditional TLC	- Lack of quantitative accuracy and reproducibility - Relative standard deviations could be ranged from 30 to 83% - Has several operating and user variables	[98–100]
Thin-layer chromatography—matrix-assisted laser desorption/ionization imaging mass spectrometry (TLC-MALDI-MS)	- Advanced version of TLC combined with mass spectrometry (MS) - The simplicity of TLC with the specific detection capabilities of MS makes it preferable compared to TLC method	- Useful for analyzing detailed patterns of PLs and LysoPLs molecular species - Allows easy visualization of all PLs with a linear range of around one order of magnitude and precision - High-throughput screening is possible - Suitable for small sample volumes	- Requires specialized equipment and expertise - The identification of unknown lipids can be challenging - Not suitable for quantitative analysis of lipid class	[101–103]
Gas chromatography with flame ionization detection (GC-FID)	- Involves a gas phase (helium/nitrogen/argon/hydrogen) and a stationary phase (narrow capillary column) - The sample is injected into a heated injected port, which is vaporized and carried by the carrier gas through the column. - The ions and electrons generated through combustion are determined and amplified by FID.	- Suitable for fatty acid detection and quantification - Highly sensitive and universal detection method of fatty acids - GC-FID can be applied to a broad range of organic compounds, from hydrocarbons and alcohols to organic acids and esters. It is especially useful for a - Provide linear response over a wide range of concentrations	- Limited sensitivity for detecting lower concentrations of FA - Not suitable for non-volatile compound - Involves a series of steps for sample preparation, resulting in a huge amount of organic solvent - PUFA or MUFA are susceptible to thermal degradation during GC analysis - Unspecific detection for all hydrocarbons, generating background noise	[104, 105]
Gas chromatography coupled to mass spectrometry (GC-MS)	- Another gas chromatography that uses a Mass Spectrometer (MS) as the detector - Suitable for complex sample matrix	- Increases sensitivity of detection and ionization compared to single MS - offers isotope enrichment information and identification of the isotope patterns of fatty acids and tracer/ratio - More powerful for compound identification compared to GC-FID - Highly sensitive and provides accurate quantification	- Expensive to purchase and needs skilled manpower to operate and maintain - Sample preparation is labor-intensive and time-consuming, especially for natural complex samples. - The biological sample can introduce the matrix effect and could interrupt the GC-MS quantification	[104, 106, 107]
Gas chromatography–combustion–isotope ratio mass spectrometry (GC-C-IRMS)	- An analytical technique used to determine the isotopic composition of carbon and, other elements (such as nitrogen, hydrogen, sulfur, and oxygen) in lipid molecules - Technique to trace the origin of a lipid in a biological system - Investigate the dietary source of lipids in humans and animals	- Suitable for quantification of labeled lipids with isotopes such as 13C and 2H - Provides results with high sensitivity and specificity - Method offering detailed analysis of lipid profiles in patient samples	- Relatively complex and expensive analytical method compared to GC-MS and GC-FID - Only used for limited element analysis, such as carbon isotope analysis. - Isotope fractionation could happen during the sample’s preparation	[108–111]
Reversed-phase liquid chromatography (RP-LC)	Highly retained hydrophilic lipids, separation based on carbon chain length and degree of FA unsaturation	- Reliable, efficient, and selective separation - Suitable for complex lipid separation - Highly sensitive and could provide accurate results at low concentrations - Could be connected with a wide range of detectors including UV-Vis, fluorescence, and mass spectrometry for quantitative and qualitative analysis - RP-LC offers excellent peak shape and high resolution, enabling the separation of complex mixtures with accuracy	- Not well-suited for the separation of highly polar compounds - Does not work properly for the separation of high-molecular-weight compounds, such as large peptides or protein - Expensive to purchase and needs skilled manpower to operate and maintain - RP-LC columns can degrade over time, leading to the loss of column efficiency and reproducibility - Some process of RP-LC uses non-volatile or semi-volatile mobile phases, which could leave residue in the mass spectrometer	[112–114]
Hydrophilic interaction liquid chromatography (HILIC)	- Suitable for analyzing polar compounds like sugars, amino acids, and glycerophospholipids - Uses a mobile phase that is typically highly aqueous - The stationary phase in HILIC is often made of materials like amino, amide, or zwitterionic phases	- Interface method between RP-LC and NP-LC - Highly effective for the separation of polar and hydrophilic compounds - Suitable for separation of LysoPLs isomers - Often provides improved peak shapes for polar compounds compared to RP-LC - Can reduce matrix effects	- Not the ideal choice for less polar or hydrophobic analytes - HILIC method development could be more challenging compared to RP-LC - The retention time of peaks is highly dependent on pH and temperature, hard to transfer the methods by keeping the same accuracy - Can introduce matrix effect - Water is the main mobile phase for this equipment, which might not be suitable for all sample types	[115–118]
Normal phase liquid chromatography (NP-LC)	- Uses a polar stationary phase and a nonpolar mobile phase - It is the opposite of reversed-phase liquid chromatography (RP-LC) - The stationary phase is polar and the mobile phase is non-polar	- NP-LC is highly effective for the separation of polar compounds, that may not be well-retained on reversed-phase columns - NP-LC can be used for sample cleanup and fractionation - Could be used for the separation of natural products	- Not suitable for the separation of non-polar compounds - Not provide accurate results for complex samples, containing a wide range of compounds with varying polarities - Extensive sample preparation is required - Like other HPLC methods, it is expensive to purchase and labor-intensive to operate and maintain - NP-LC column has a shorter lifespan than other columns	[119, 120]
Nuclear magnetic resonance (NMR) spectroscopy	- A spectroscopic tool used for analyzing lipids from the natural complex matrix - This can be configured for the analysis of various isotopes, 13C, 31P, 15N, 1H - Used for structure-specific analysis of fatty acids	- Provide both detection and quantification—has a wide range of applications - Can be used to investigate fatty acid composition, lipid class, phospholipid composition - Non-destructive and non-invasive method - Sample preparation is relatively easy - The same sample could be run under different isotopic labeling including 13C, 31P, 15N, and 1H NMR - NMR spectra are highly reproducible and provide accurate results for qualitative and quantitative analyses	- Expensive to purchase, operate, and maintain - Need higher quantity for the compound with low concentration - NMR spectra could be affected by buffer pH and temperature, resulting in hard-to-assign peaks - Provide limited resolution for complex compound - NMR spectra require long acquisition time for higher scan number - NMR data analysis is more complex than chromatographic data - Require strict safety precautions, as it is operated under a high magnetic field	[121–128]
Raman spectroscopy	- Suitable for analyzing FA degree of unsaturation in storage lipids of lipid droplets in biological samples - provides information about the chemical composition, molecular structure, and physical properties of substances - Raman spectroscopy is based on the Raman scattering phenomenon	- Non-destructive and non-invasive method - Can quantify omega-3 fatty acids from outside of the fish oil/krill oil capsule - Less labor-intensive - Involves little to no toxic chemicals, making it a more eco-friendly approach than chromatographic techniques - Provides qualitative information and a specific fingerprint for each lipid, offering insights into intra- and inter-molecular vibrations and enhancing the understanding of a reaction - Requires minimal or no sample preparation	- Less accurate in the quantification of fatty acids than chromatographic methods - High background noise due to impurities - Data analysis is complex and requires specialized software for chemometric analysis - Not sensitive to polar covalent bonds (e.g., O–H, N–H, C=O) - Affected by water interference	[129–135]
Fourier-transform infrared spectroscopy (FT-IR)	- Another type of vibrational spectroscopy, which also could identify and quantify phospholipids depending on the vibration of molecules in biological samples	- IR spectroscopy is particularly sensitive to polar covalent bonds (e.g., O–H, N–H, C=O) - IR spectroscopy is less affected by water interference - Non-destructive and non-invasive method - Require less or no sample preparation	- Provide less structural information or less efficient in distinguishing between structural isomers - Produce overlapping peaks, making it challenging to identify specific functional groups or components. - IR spectroscopy is less sensitive to non-polar compounds - The thickness of the sample can affect the intensity of IR absorption bands	[112, 136] [134, 135]
ESI-MS (electrospray ionization MS)	- An advanced analytical technique that could offer not only qualitative (structure) but also quantitative (concentration or molecular mass) information on analyte molecules - Molecules are first introduced into the ionization source and then the ion travel through the mass analyzer and make contact with the detector - Finally, the computer displays the signals graphically	- Broad applicability for lipid classes. ESI-MS can analyze a wide range of compounds, including small organic molecules, peptides, proteins, nucleic acids, lipids, carbohydrates, and various biomolecules - ESI-MS is highly sensitive and capable of detecting analytes at low concentrations, even in complex mixtures - ESI is considered a “soft” ionization technique, meaning it causes minimal fragmentation of analyte ions during the ionization process - ESI can operate in various ionization modes, such as positive and negative ionization, allowing flexibility in analyzing different types of compounds	- Sensitivity to contaminants means the ionization could be affected by contaminants and the quality of mass spectra - Expensive to purchase and complex to operate and maintain - Require liquid samples, meaning solid and volatile samples can be challenging to analyze - Complexity in resolving isomers - Potential ion suppression effects - Suppression of ionization signals caused by matrix interference. When analyzing a biological sample, the ionization signals obtained are notably lower in comparison to those achieved with pure standard solutions containing equivalent analyte concentrations - One serious disadvantage of internal calibration is the risk of calibrant signals overlapping analyte signals	[137–140]
MALDI-MS (matrix-assisted laser desorption/ionization MS)	- Uses laser desorption/ionization with a matrix to analyze the solid lipid samples - Could be both silver or gold-assisted ionization imaging mass spectrometry	- Suitable for large lipids and lipid imaging - High specificity and sensitivity can be obtained for cholesterol, triglycerides and fatty acids - MALDI-MS can be used for high-throughput screening due to its rapid data acquisition - Gentle ionization process minimizes the sample degradation, preserving the integrity of biological molecules.	- Matrix interference for small lipid species. The presence of interfering matrix ion peaks below 800 Da interferes with the detection of small molecules - Limited for small, volatile lipids - MALDI-MS is primarily designed for solid and liquid samples and may not be suitable for analyzing gaseous samples	[141–143]
QQQ-MS (triple quadrupole MS)	Involves the use of three quadrupole mass analyzers arranged in series to selectively filter ions based on their mass-to-charge ratios, allowing for precise quantification and structural elucidation of target analytes (such as lipids) in complex mixtures	- Precise and quantitative lipid analysis including lipid class separation and species identification - It can differentiate between multiple analytes, even when they have similar mass-to-charge ratios - Excellent sensitivity for lipid quantitation - QQQ-MS is less susceptible to matrix effects compared to some other mass spectrometry techniques	- Limited structural information.ires specific lipid standards for quantification - Expensive to purchase, complex setup, operate, and maintain - Developing robust and optimized QQQ-MS methods can be time-consuming and may require significant effort - QQQ-MS may struggle to differentiate between structural isomers, as their mass-to-charge ratios may be identical	[144, 145]
LC-MS (liquid chromatography–mass spectrometry)	Combines liquid chromatography with mass spectrometry for separation and analysis of lipid classes	- Separation and identification of diverse lipid species - Highly sensitive and capable of detecting and quantifying compounds at very low concentrations - Modern LC-MS instruments offer high mass accuracy and resolution - Suitable for accurate determination of molecular weights and the identification of complex molecules - Can cover broad lipid coverage and is suitable for quantitative lipidomics	- LC-MS instruments can be complex and require expertise for operation, maintenance, and data analysis - susceptible to matrix effects - - preparation involves a series of extraction and purification steps, which can lead to the use of toxic organic compounds - Some LC-MS instruments may have a limited mass range, which can restrict their applicability for high-mass or very low-mass compounds. - Method development is laborious and requires extensive optimization to achieve reliable and reproducible results	[106, 146, 147]
MS/MS (tandem mass spectrometry)	Employs two or more mass analyzers in series for structural elucidation and quantification of lipids	- Structural characterization of lipid species - High sensitivity for lipid identification - Enhanced specificity compared to single-stage mass spectrometry techniques - MS/MS is less susceptible to matrix effects compared to other MS - Suitable for the detection of trace-level compounds even in the presence of high-abundance species. - Can be configured for high-throughput analysis - Can be used for non-targeted analysis	- Complex setup with longer analysis times - Method development and standardization are difficult - Interpretation of MS/MS spectra can be challenging, and automated data analysis software may not always provide accurate results - MS/MS may require larger sample amounts compared to other techniques	[148–151]

By combining several techniques, researchers can obtain a comprehensive analysis of LysoPLs and PLs and their roles in neurological diseases’ underlying mechanisms of disease, and inform about the development of new treatments. In this section, we will discuss each analytical technique highlighting their principles, applications, and recent advancements in phospholipidomics.

### Chromatographic Techniques in Phospholipidomics

#### Thin-Layer Chromatography (TLC)

Thin-layer chromatography (TLC) has been a widely used and cost-effective technique for separating phospholipids (PLs). Over the years, various methods have been developed to optimize the separation of different species of PLs and LysoPLs from biological matrices [93].

Automated high-performance thin layer chromatography (HPTLC) has been considered an improved version of TLC offering high elution of molecules and generating less background noise than classical TLC due to the silica particle size of the stationary phase [96]. However, eluting highly polar LysoPLs from silica gel requires organic solvents with high polarity.

Although TLC has been suitable for separating PLs and LysoPLs, its preparative applications have been limited. In specific cases of PUFAs, long-term storage of TLC plates resulted in lipid oxidation. On the other hand, TLC became an unusual approach for PLs and LysoPLs quantification in case it is combined with other chromatographic techniques such as GC (TLC-GC) [98]. Also, TLC can be coupled to mass spectrometry as an emerging lipidomics approach for analyzing detailed patterns of PLs’ and Lyso-PLs’ molecular species such as TLC-blot-matrix-assisted laser desorption/ionization imaging mass spectrometry method (HPTLC-MALDI-MS) [101, 102]. This approach has separated PL mixtures directly from the HPTLC plate. Furthermore, it has allowed easy visualization of all PLs with a linear range of around one order of magnitude and precision, making it useful for differential analysis of lipids [101, 102].

### Gas

#### Gas Chromatography (GC)

In lipid profiling, GC has been ideally suitable for FA analysis. Indeed, volatile fatty acid methyl esters (FAMEs) have been produced through derivatization [152]. GC with flame ionization detection (GC-FID) has been the common GC in lipidomics based on FAMEs’ retention time. Organic compounds have been combusted into a hydrogen flame and molecules became thermally ionized for detection.

FA regioisomerization cis/trans separation has been also possible through GC-FID although several drawbacks [52, 153]. Some of these have been related to the limitations of sensitivity for the detection of lower concentrations of FA and that FID has been unspecific to FA but for all hydrocarbons. Consequently, the non-specificity of FID has increased the background generated from hydrocarbon contamination hence decreasing the technique’s sensitivity. Recently, short-chain SFAs such as 10:0 and 13:0 were not detected [152]. Thus, in the context of lipid profiling, GC-FID had a limitation to the detection of long-chain FAs principally.

To overcome GC-FID’s limitations, GC coupled to MS (GC-MS) has been considered as a valuable technique increasing the sensitivity of detection and ionization compared to a single MS [154]. In GC-MS, electron ionization (EI) has been the ionization source frequently used where an electron beam ionized sample molecules resulting in electron loss. Indeed, to ensure high fragmentation of lipids, a high-energy ionization pathway was needed. This ionization procedure has been destructive for charged molecules and qualified as a “hard” source of ionization. Contrarily, electrospray ionization (ESI) or atmospheric pressure chemical ionization (APCI) have been “soft” sources and are only compatible with HPLC. Nevertheless, GC-MS has offered information about isotope enrichment by identifying the isotope pattern of FAs and tracer/ratio [155].

For stable isotopic analysis, gas chromatography combustion isotope ratio mass spectrometry (GC-C-IRMS) has been the common approach. For optimal separation of labeled FAMEs, GC-C-IRMS has been more suitable for quantification of labeled lipids with isotopes such as ^13^C and ^2^H. GC-C-IRMS has remained the best approach in clinical trials based on supplementation of lipids labeled with stable isotopes [156].

Indeed, clinical lipidomics can benefit greatly from GC-C-IRMS to detect and quantify lipids in biological samples such as blood, serum, and plasma. GC-C-IRMS has been recognized for its high sensitivity and specificity, making it effective in detecting changes in lipid levels that may indicate certain diseases.

The potential of GC-C-IRMS in revolutionizing clinical lipidomics lies in its ability to provide accurate and detailed analysis of lipid profiles in patient samples. This capability can help in the diagnosis and management of various diseases by providing a full understanding of the patient’s lipid profile [157–159].

Furthermore, this method can offer researchers precise and dependable data to explore the metabolism of labeled lipids in vivo, which could explain the mechanisms involved in lipid metabolism and develop new therapeutic interventions. Despite GC-IRMS’s promising results, further research is required to validate its application in clinical lipidomics and establish standardized protocols for sample preparation and analysis. Nevertheless, considering its accuracy and reliability, GC-IRMS is crucial for investigating the role of lipids in metabolic processes and developing innovative strategies to improve brain health.

#### High-Performance Liquid Chromatography (HPLC)

HPLC has been a highly reliable, efficient, and selective separation method widely used in lipidomics. Different HPLC systems can be distinguished based on columns’ characteristics including the particles’ size, the column’s length, as well as the composition of the mobile phase.

Reversed-phase liquid chromatography (RP-LC) has been frequently chosen for complex lipid separation because hydrophilic lipids are highly retained and their separation depends on carbon chain length and degree of FA unsaturation [160, 161].

Moreover, hydrophilic interaction liquid chromatography (HILIC) mode has been developed to retain polar compounds like sugars, amino acids, and glycerophospholipids. HILIC is an interface method between RP-LC and NP-LC with a stationary phase identical to that of NP-LC (Si, NH_2_, amide, diol…) and a mobile phase similar to that of RP-LC with organic solvents immiscible with water like acetonitrile. More recently, researchers could separate LysoPLs isomers by using a normal phase column by HILIC-ESI-MS/MS [159].

Furthermore, a versatile method to separate LysoPL isomers (1-acyl-2 LPLs and 2-acyl-1 LPLs) and prevent acyl migration reaction in LysoPLs was implemented through a simple procedure involving specific pH and temperature conditions. In this study, pH and temperature effects on the enzymatic reaction with a phospholipase A1 (PLA1) from *Rhizomucor miehei* lipase were investigated. This newly developed method is of great importance to the lipidomics field since LysoPLs play a crucial role in various physiological processes, such as inflammation and neurodegeneration. The ability to selectively separate and analyze different isomers of LysoPLs with HPLC-MS/MS can provide researchers with valuable insights into the underlying mechanisms of these processes [162].

Regarding the choice of detector with HPLC for efficient lipids separation and identification, ultraviolet (UV) detector or evaporative light scattering detector (ELSD) are common detectors. Indeed, LC-ELSD is one of the most suitable applications to quantify PLs in different food matrices [157, 158]. LC-UV is frequently used for lipids analysis and is recommended for PLs and LysoPLs separation and quantitation. Researchers developed a method to isolate LysoPLs from PLs in one single run and used a balance study approach [163–166]. The robust interpretation of altered PLs levels observed in pathologic states requires the ability to assess recovery by lipid phosphate balance, identify LysoPLs, quantify unexpected organic phosphorous-containing compounds, and consider alterations in acyl-group content or composition via the acyl/organic phosphate ratio [167–169].

Furthermore, for more complex lipids, two-dimensional liquid chromatography (2D-LC) is suitable to separate lipids following independent parameters such as electrostatic force, hydrophobic character, size exclusion, ion charge, and affinity [170]. The first dimension separates lipid classes by NP-LC or HILIC and the second dimension is analyzed by RP-LC. Conversely, this system is time-consuming and not easy to calibrate [171]. More recently, for TAG identification in adults’ and infants’ formula analysis, three-dimensional liquid chromatography (3D-LC) was applied. High-quality separation and TAG identification were achieved through coupling 3D-LC with MS [162].

### Spectroscopic Methods in Phospholipidomics

#### NMR, Raman Spectroscopy, and FTIR

Spectroscopy aims to study the interaction of electromagnetic radiation with substances. Several spectroscopic methods have been applied in lipidomics including nuclear magnetic resonance (NMR) spectroscopy, Raman spectroscopy, and Fourier-transform infrared spectroscopy (FT-IR).

NMR has been a useful method in lipidomics investigating lipid structural features through natural isotopes such as ^31^P, ^1^H, or ^13^C pre-enriched lipids. In vivo, lipids’ metabolism in the liver has been investigated through NMR analysis [125]. As reported in numerous studies, NMR has many applications in determining lipids’ metabolism in pathogenesis as well as lipids’ interaction with proteins, drugs, or antibiotics [172, 173]. Through NMR, the interaction between polyphenols, salivary proteins, lipids in food, and oral membrane taste receptors during wine tasting has been identified [174]. To surpass the abundant drawbacks of spectral overcrowding when recording 1D NMR spectra on such samples, the acquisition of two-dimensional 2D NMR spectra has permitted an enhanced separation among coincided resonances while yielding precise quantitative information [175]. In lipidomics, NMR had several advantages over other spectroscopic techniques such as qualitative results and a vast range of applications. NMR can study the lipids’ structure and their derivatives’ structure as well. The interaction of lipids within proteins in cell membranes has also been reported [93]. Moreover, NMR has been an interesting technique to illustrate the structure of complex mixtures of lipids in the food technology and nutrition field [176]. In phospholipidomics, 31P NMR spectroscopy has provided a quantitative and fast approach to characterize lipids in cell membranes with highly predictable and reproducible results [177, 178]. Recently, a simple method to quantify LysoPLs in food emulsions by 31P NMR, a sensitive and precise method of quantification of LysoPLs has been suggested [179]. Moreover, in lipidomics, NMR has been mostly combined with mass spectrometry approaches to reach a complete and robust PL profile [180–183].

Another spectroscopic approach applied in phospholipidomics has been Raman spectroscopy, a molecular spectroscopic system founded on the interaction of light with matter or a light scattering process to provide data about a material’s characteristics. This spectroscopic technique has provided information about intra- and inter-molecular vibrations and an additional understanding of a reaction. For lipids’ analysis, Raman spectroscopy has offered qualitative information, considered as a specific fingerprint for each lipid, including the degree of unsaturation, cis/transposition, and chain length. Researchers have suggested lipids profiling using multimodal approaches by combining Raman spectroscopy and mass spectrometry to improve the analysis of brain pathologies. During analysis, mass spectrometry has been reported to be more sensitive in detecting cholesterol ester than Raman spectroscopy. However, the use of single-cell laser trapping combined with Raman spectroscopy has remained a suitable option to optimize qualitative results on the level of unsaturation and transition temperatures of lipid species [129]. Thus, it has been reported that this spectroscopic technique is suitable for analyzing FA degree of unsaturation in storage lipids of lipid droplets in algae and liver disease [130–132].

Additional spectroscopic approaches included Fourier-transform infrared spectroscopy (FT-IR) based on light’s interaction with matter to gain insight into matter’s functions relying on the absorption of light. In lipid profiling, FT-IR has characterized the acyl chain length of FAs as described by Stoll et al. [136]. These researchers have observed with FT-IR that short-chain FAs in PC affected the confirmation of the red blood cell membranes, unlike long-chain FAs [136].

#### Mass spectrometry, High-Sensitivity Techniques in Phospholipidomics

Mass spectrometry (MS) is a state-of-art analytical approach, which allows fast and reliable identification and quantification of lipids in lipidomics for biomedical and biochemical research applications [184].

MS-based phospholipidomics has offered several advantages over traditional methods such as TLC and HPLC. This technique has afforded higher sensitivity, specificity, and accuracy in lipid detection and quantification. With MS, even low-abundance lipids in complex biological matrices can be identified and characterized with high precision and resolution. These features are essential for novel lipid species discovery and the elucidation of their roles in biological processes [185].

MS became the analytical technique of choice for several omics branches and phospholipidomics is one of these [186]. All MS-based omics approaches have followed the same steps from sample preparation to MS spectra analysis. The first step is the sample separation followed by sample analysis using a separation technic such as LC, GC or capillary electrophoresis (CE), or supercritical fluid chromatography (SFC). Mass spectrometric measurements have been achieved through diverse ionization methods such as electrospray (ESI), electron ionization (EI), desorption electrospray ionization (DESI) for “matrix-assisted laser desorption and ionization” (MALDI). Different ions have been separated and detected depending on their m/z values in the mass analyzer. The final stage consisted of MS spectra storage. Signal intensities have been proportional to the molecular species’ abundance [186].

To accurately quantify lipids using MS, internal standards (IS) have been utilized to account for variable recovery from biological matrices and other factors that may affect ion yield. Generally, IS had similar structural, ionization, and fragmentation properties as the lipid classes being analyzed [187]. Synthetic non-endogenous PLs have been commonly used as internal standards to quantify PLs in biological samples and estimate extraction yield. For additional validation and quantification of a specified molecule in targeted lipidomics, particular precursor m/z values and select probable fragment m/z values have been traced via vigorous triple quadrupole MS tools in selective reaction monitoring (SRM) mode allowing lipid identification and quantitation on the class level.

In targeted lipidomics, to accomplish a deeper MS fragment analysis and allow species and subspecies identification, orbitrap-type or time-of-flight (TOF) MS instruments with advanced mass resolution can achieve a full-scan acquisition in parallel reaction monitoring (PRM) mode for particular precursors, evaluating all fragment ions instantaneously [188].

To investigate lipids’ structure, tandem mass spectrometric tests (MS-MS) have been applied in lipidomics and numerous fragmentation procedures such as collision-induced dissociation (CID) have recorded precursor precise fragment spectra [189].

Furthermore, in MS lipids analysis, to identify molecules, a special software has compared the generated MS-MS spectra with theoretical fragment spectra or with reference spectra from a database [188].

Higher-level fragmentation for lipids’ identification and quantification has been also relevant where a mass spectrometer picks MS2 fragment ions for additional fragmentation (MSn) producing MSn spectra. All collected data with chromatographic retention time, drift time, collisional cross-section, scan polarity, collision energies, and relevant metadata have been stored in specific database [188].

Several lipidomics databases have been available. Among these, LIPID MAPS is a relational database for structures and annotations of biologically related lipids comprising lipid classification, experimentally determined structures, in-silico combinatorial structures, and other lipid resources [190]. LipidHome and Swiss Lipids are also databases in lipidomics providing respectively the in-silico generated theoretical lipid structures and curated database of lipid structures with experimental evidence and integration with biological knowledge and models [191, 192].

A number of advanced dedicated software for lipid identification from mass spectrometry are also accessible. Hoffman et al. [188] reviewed a total of 31 available software tools for lipidomics data processing and identification published between 2006 and 2021. Several software such as LIMSA, LipidomeDB, LipiDex, LipidHunter, LipidMatch, Greazy, LipidMS, LipoStar, LipoStarMSI, LPPTiger, and Lipidview were included. Some software including LipidHunter and Greazy supports phospholipids only whereas others support oxidized phospholipids only (LPPTiger).

As previously mentioned, several ionization modes have been developed for lipids’ analysis in biological samples. Among these, ESI is the common ionization source for lipids’ identification in plasma [192]. To identify plasma lipoprotein-linked phospholipids, Dashti et al. [193] applied three approaches including LC-ESI/MS, LC-ESI-MS/MS, and HPTLC analysis of diverse lipoprotein portions collected from pooled plasma. PE, PI, and SM were only found on lipoproteins whereas PC and LysoPC were associated with both lipoproteins and plasma lipoprotein free fraction (PLFF). Authors have suggested that LC-ESI-MS/MS has been the greatest approach for evaluating the lipids’ content of biological samples such as the PL composition of plasma lipoproteins [193]. Khedr et al. [194] who investigated serum PL profiles of healthy volunteers and patients with newly diagnosed dengue fever (DF), hepatitis B (HBV), and hepatitis C (HCV) have suggested the same conclusion. The research team has followed an approach for the characterization and quantification of potential PLs biomarkers in human serum by means of LC-ESI-MS/MS and a non-endogenous PL mixture as an internal standard [194]. For the characterization and quantification of PLs, two ESI-MS-MS have been utilized. respectively: ion trap mass spectrometer (IT-MS) and triple quad mass spectrometer (TQ-MS). Each MS system has been linked to an HPLC system. PC, PI, PE, and PS have been characterized in human serum using LC-IT-MS. Lyso-PCs have been also identified. Through the analysis of major serum PLs in healthy volunteers and three groups of viral infection cases, different lipid profiles have been identified in patients with viral infectious diseases in comparison to healthy subjects reflecting the disrupted lipid metabolism in diseases [194].

Other than ESI, desorption electrospray ionization (DESI), an ambient system functional in mass spectrometry (MS), has permitted for an in situ analysis of PLs with few to no sample pretreatment [195]. One more advantage of DESI-MS has been the direct surface sample analysis in phospholipidomics [196]. More recently, phospholipidomics analysis (LysoPE, LysoPC, SM, PA, and PC) of blood samples has been achieved through PARAFILM-based dried plasma spot (DPS) sampling and DESI-MS method [197]. Additionally, matrix-assisted laser desorption ionization (MALDI) has been convenient for lipids profiling of neutral lipids.

Despite the high sensitivity of MS, it had some limitations compromising the interpretation of data. One of these limitations has been linked to the complex mixture’s resolution containing isobaric and isomeric lipid species and requiring caution for automatic data processing from online databases. This limitation can be overcome by implementing three approaches. The first one consisted of using highly efficient chromatography such as the HILIC-based method [198]. The second approach has been established on the use of a shotgun-like with differential mobility spectrometry (DMS) allowing a good separation of the lipids’ classes in the gas phase before analysis by MS [184, 199]. The last option has been considered using high-resolution accurate mass spectrometry (HRAMS) capable of high accuracy (2 ppm) and can typically identify molecules at the sum composition level depending on instrument type [162, 200].

Indeed, hydrophilic interaction chromatography coupled with electrospray ionization mass spectrometry (HILIC-ESI-MS) has been implemented for phospholipidomics [198, 201]. HILIC-ESI-MS has been an operational method employing the hydrophilic stationary phase with reversed-phase eluent to isolate and analyze numerous lipid molecules. When coupled with MS, the HILIC mode has gained significant sensitivity improvement. For the polar molecules’ acid charges such as Lyso-PS and Lyso-PA, the HILIC system has been recommended [202]. This system has been designed for the separation and purification of polar compounds such as PLs based on hydrophilic interaction hydrogen bonding and weak electrostatic interactions [115, 159, 203].

In addition to HILIC-ESI-MS, Hydrophilic Interaction Chromatography coupled with quadrupole ion trap mass spectrometry (HILIC-QTRAP-MS) has been used to phospholipidomically examine swimming crabs, *Portunus trituberculatus*, cultivated with formulated feed, frozen trash fish, and mixed feed. Four PL classes involving 81 molecular types with numerous PUFA have been identified. Results showed that the formulated feed group retained the utmost concentration of PL (332.91 μg·mg^−1^), afterwards frozen trash fish group (294.74 μg·mg^−1^) and mixed feed group (279.74 μg·mg^−1^). Phospholipidomics outcomes showed that formulated feed might substitute frozen trash fish for the cultivation of *P. trituberculatus* [204].

Finally, mass spectrometry imaging (MSI) technics have emerged in lipidomics providing complementary results about lipids’ composition and distribution in the brain. In terms of techniques, innovative lipidomics approaches, such as mass spectrometry imaging (MSI), can offer insight into the role of lipids in brain function and disease response [205–207]. More recently, atomistic simulations have compared lipid bilayers with complex and varied human brain compositions, leading to the discovery of precise ranges for lipids’ head groups, tail lengths, saturation, symmetry, and asymmetry [208]. These findings have the potential to further explore the fundamental role of the lipid bilayer in the permeability and transport of small molecules across the blood-brain barrier (BBB).

This approach has provided complementary data about the diversity and dynamics of lipids in the brain and other organs to extend the understanding of biochemical changes in an organism’s function [209].

## Next-Generation Tools for Lipidomics and Future Directions

Next-generation lipidomics is the future of cutting-edge research, enabling in-depth quantitative and qualitative analysis of lipid samples while minimizing the impact of sample matrix, all within a shorter timeframe, and with higher sensitivity and accuracy. Sophisticated bioinformatics tools and databases are necessary for processing a large volume of lipidomics data, enabling the identification and automation of the quantification system for the discovery of meaningful lipid biomarkers.

Over the last two decades, the development of bench-top, user-friendly mass spectrometers has expanded our knowledge of biochemistry and lipidomics analysis [210]. The combination of HPLC or UPLC with electron spray ionization (ESI) made comprehensive lipidomics analysis accessible to new generations of researchers. This combination offers high selectivity, specificity, and accuracy that was previously out of imagination. LIPID MAPS, which stands for the lipid metabolites and pathways strategy, facilitated the development of resources and methods, serving the next generation of lipid researches with tools, resources, data, and training [210]. The uses of LIPID MAPS started during the development of MS methods, generating internal standards labeled with isotopes, analyzing macrophases, and sharing data for global research communities [211]. Recently, scientists are investigating in a very different way. Younger scientists are examining lipids in a holistic manner rather than identifying and quantifying individual lipid classes or species, which is in agreement with the concept of developing system biology [210]. However, a basic understanding of lipid biology and biochemistry is always required, if we want to decipher new data and investigate the power of lipidomics.

Another revolutionary force in lipidomics is imaging mass spectrometry (IMS), which offers high molecular specificity, sensitivity, and the spatial distribution of small molecules in tissues [212, 213]. Advanced techniques such as matrix-assisted laser desorption/ionization imaging mass spectrometry (MALDI-IMS) and secondary ion mass spectrometry (SIMS) facilitate to researchers the visualization of lipid distributions within tissues and individual cells [214, 215]. Emerging techniques in single-cell lipidomics and imaging have introduced infinite opportunities for the analysis of lipid heterogeneity [216]. The latest advancement in lipidomics analysis is the adoption of high-throughput techniques, significantly expediting the speed of lipidomics research. These techniques enable the efficient and reliable analysis of extensive sample sets. Robotic platforms and automation systems have simplified sample preparation and analysis, making lipidomics data more accessible to the broader research community. In clinical research, advanced lipidomics tools are reported for the identification of specific lipid biomarker profiles, which helps the diagnosis and management of a wide range of diseases including PD, AD, schizophrenia, and various cardiovascular conditions, as mentioned earlier in Table 1. Lipidomics could also help in drug development and personalized medicine by tailoring treatments to individual lipid profiles [36]. However, there are several challenges, including data acquisition, integration, standardization, and the overall development of lipid identification algorithms, in lipidomics research. Future directions in lipidomics research could be the integration of multi-omics approaches, which will help to understand the role of lipids in biological samples.

Overall, next-generation lipidomics technologies have a greater impact on healthcare and enhance our knowledge on understanding neurodegenerative diseases. Researchers can predict much more profound understandings of lipid-related illnesses based on the distribution of lipid biomarkers. Furthermore, next-generation lipidomics techniques help to create innovative treatment approaches with the development of numerous technologies including mass spectrometry, liquid chromatography, bioinformatics, imaging, and single-cell methods, making it a crucial and dynamic area of biomedical research.

## Conclusion

In the human brain, lipids play fundamental roles, and their composition could be altered with age and in various neurological diseases. Lipidomics, an essential tool highlighting the mechanisms involving lipids in health and diseases, could identify lipid biomarkers for diagnosis and therapy. More specifically, in the brain, the identification of specific lipid species as biomarkers for different brain disorders, such as AD, PD, schizophrenia, bipolar disorder, and epilepsy could support the medical sector in the development of targeted therapies. Indeed, phospholipidomics is a cutting-edge analytical technique that holds immense potential for investigating the mechanisms and potential therapeutic targets of neurological diseases. By comprehensively analyzing PLs’ profile of patients with neurological disorders, researchers can gain valuable insights into cerebral molecular alterations, potentially leading to the development of more effective treatments. Furthermore, combining phospholipidomics with other analytical techniques, such as proteomics and genomics, might offer a more comprehensive understanding of the complex molecular pathways involved in neurological diseases. This integrated approach can assist in the identification of novel biomarkers and therapeutic targets that have been overlooked via a single analytical method. Combining multiple analytical approaches for lipidomics analysis, the complexity of lipids’ metabolism in the brain, and the lack of systematic lipid databases have made the interpretation of lipidomics data challenging. Therefore, further research is needed to fully elucidate the role of lipids in neurological diseases and establish the clinical utility of lipidomics analysis. In addition, the standardization of sample collection, processing, and analysis is critical to ensure the reproducibility and comparability of lipidomics data across studies. Despite these challenges, lipidomics has already yielded important discoveries, such as the role of specific lipid species in neuroinflammation and neuronal cell death. Additionally, the integration of lipidomics with other omics approaches, such as genomics and proteomics, can provide a full understanding of the molecular mechanisms underlying neurological disease biomarkers in affected brain areas and suggest therapeutic mechanisms to transport vital lipids to the brain.

Finally, this review serves as a valuable resource for researchers and clinicians alike, providing a concise summary of the current knowledge on phospholipidomics in neurological diseases and highlighting areas that require further investigation. By promoting collaboration and knowledge sharing across different disciplines, we can accelerate the pace of discovery and ultimately improve the lives of patients suffering from neurological disorders.

## Data Availability

Not applicable.
